# Obituary

**Published:** 1908-04-15

**Authors:** 


### OBITUARY.

THOMAS FILLEBROWN, M.D., D.M.D. The death of Professor Fillebrown, which occured January 22, 1908, at the Boothby Hospital, Boston, removes one of the most prominent and best known New England dentists.

Thomas Fillebrown was born January 13, 1836, at Winthrop, Maine. He was the son of James Bowdoin and Almira Butler Fillebrown. He received his early education in the public schools, the Towle Academy and Maine Wesleyan Seminary, from which he graduated in 1859. In this institution he became a teacher of higher mathematics, later studying dentistry with his father, Dr. J. B. Fillebrown. He studied medicine at Bowdoin College in the medical school of Maine, from which he received the M. D. degree in 1863. Desiring to further equip himself, he entered the first class of the Dental Department of Harvard University, and graduated from this department in 1869 as D.M.D. He practiced first at Lewiston and Portland, Me., and after his graduation in dentistry located in Boston, where he became an instructor in the Dental Department of Harvard University. Later he was elected Professor of Operative Dentistry and Oral Surgery, a position he held for twenty-one years.

Dr. Fillebrown made many valuable contributions to dental literature. His best known work was the Fillebrown Text-book on Operative Dentistry. He also wrote for dental journals many articles on the subject and on oral surgery, hare-lip, cleft palate, hypnosis as a dental obtundant, anesthesia, and lately several papers on the physiology of vocalism. He was in demand as a lecturer before surgical and dental associations. He was a faithful and active society attendant. Following the merging and consolidation of the American Dental Association and the Southern Dental Association, Dr. Fillebrown was elected the first president of the present National Dental Association at Old Point Comfort, August 5, 1897. He served in this capacity at the next session of the association held in Omaha, August 30, 1898. He was also president of the Massachusetts State Dental Association, president in 1907 of the American Academy of Dental Science at Boston, a member of the Maine State Dental Society, the American Medical Association, the Massachusetts Medical Society and the Maine Medical Association.

In 1862 he married Miss Helen Dalton, of Kenfield, Me. To them were born Edith S., who died in infancy; Harriet A., Helen F., Dr. Charles D. and Winthrop Fillebrown, who survive him. The funeral services were held at the residence of Dr. Fillebrown's brother, C. B. Fillebrown, on Friday, January 24, and the remains were interred at Portland, Me. Dr. Fillebrown's work in operative dentistry and oral surgery, at which he was an expert, will stand out prominent for many years to come. His life was one of devotion to his profession and his influence as a teacher and writer leaves a record of good work and devotion for his chosen calling.—Dr. Thorpe in March *Brief*.

